# Acute Coronary Syndrome in Pregnancy and the Post-Partum Period

**DOI:** 10.3390/jcdd9070198

**Published:** 2022-06-23

**Authors:** Anna C. O’Kelly, Jonathan Ludmir, Malissa J. Wood

**Affiliations:** Division of Cardiology, Massachusetts General Hospital, 55 Fruit Street, Boston, MA 02114, USA; aokelly1@mgh.harvard.edu (A.C.O.); jludmir@mgh.harvard.edu (J.L.)

**Keywords:** acute coronary syndrome, pregnancy-associated myocardial infarction, shock, cardio-obstetrics team

## Abstract

Cardiovascular disease is the leading cause of maternal mortality in the United States. Acute coronary syndrome (ACS) is more common in pregnant women than in non-pregnant controls and contributes to the burden of maternal mortality. This review highlights numerous etiologies of chest discomfort during pregnancy, as well as risk factors and causes of ACS during pregnancy. It focuses on the evaluation and management of ACS during pregnancy and the post-partum period, including considerations when deciding between invasive and non-invasive ischemic evaluations. It also focuses specifically on the management of post-myocardial infarction complications, including shock, and outlines the role of mechanical circulatory support, including veno-arterial extracorporeal membrane oxygenation (VA-ECMO). Finally, it offers additional recommendations for navigating delivery in women who experienced pregnancy-associated myocardial infarction and considerations for the post-partum patient who develops ACS.

## 1. Introduction

Cardiovascular disease (CVD) is the leading cause of maternal mortality in the United States [1]. Recent data suggest ischemic heart disease, which accounts for <2% of cardiovascular disease in pregnancy [2,3], and pregnancy-associated myocardial infarction (PAMI), are leading causes [1,2,3,4]. Acute coronary syndrome (ACS) is 3 to 4 times more likely to affect women during pregnancy than similarly aged, non-pregnant women [5], and is estimated to affect 3–10 of every 100,000 pregnancies [5,6,7,8,9,10]. MI during pregnancy is also associated with higher mortality than in non-pregnant women [5,8]. The incidence of PAMI is especially high in the United States, with data showing it is at least four times more common than in both Canada and Europe [10]. Though the cause of these differences is not entirely clear, and is likely multifold, potential contributors include a higher burden of risk factors in US-based populations, delays in care associated with insurance barriers, and limited outpatient wraparound care [10,11]. The maternal mortality rate in the US is 5–11%, which is higher than in Canada and Europe [5,7,10,12]. Unfortunately, the rates of ACS in pregnancy are rising [5,7,9,10,13] and are expected to continue to rise [14,15]. Early recognition of ACS in pregnancy is essential to facilitate optimal management.

## 2. Chest Discomfort in Pregnancy

There are many etiologies of chest pain in pregnancy. ACS mimickers that occur outside of pregnancy, including pulmonary embolism, myocarditis, pericarditis, Takotsubo cardiomyopathy, and aortic dissection, can also occur during pregnancy. Nevertheless, ACS should always be considered and must be excluded. This is especially true given the higher risk of MI during pregnancy [5].

## 3. Causes of ACS in Pregnancy

ACS can affect women at any stage during their pregnancy, though it is most common in the late third trimester and early post-partum period [10,16]. A large retrospective study found that the number of women with PAMI in the post-partum period was more than double that of women in the antepartum period (53.5% versus 20.6%, respectively) [16]. ACS in pregnancy, just as in the non-pregnant state, can be caused by both obstructive and non-obstructive coronary disease. Unlike in the general population, however, the majority of cases of ACS in pregnancy are caused by non-atherosclerotic etiologies, and in those with atherosclerosis with non-obstructive lesions [8,17]. Myocardial infarction with non-obstructive coronary arteries (MINOCA), which accounts for 5–6% of acute MI in all-comers, tends to be more common in younger, female patients [18]. Many of the most common mechanisms of ACS in pregnancy, including coronary vasospasm and coronary thrombosis and embolism, are common causes of MINOCA. The mechanism of PAMI may also vary by pregnancy period, as coronary artery disease and coronary thrombosis may contribute disproportionately to antepartum PAMI, whereas SCAD is most common in post-partum PAMI [8]. The variability in these data is likely driven by limited sample size and study numbers.

### 3.1. Spontaneous Coronary Artery Dissection

Spontaneous coronary artery dissection (SCAD) accounts for an estimated 14–43% of pregnancy-associated MI [5,8,12,16] and is the most common cause of ACS in pregnancy. SCAD is caused by a spontaneous dissection of one or multiple coronary arteries leading to an obstructive hematoma. Many of the physiologic and hemodynamic changes in pregnancy are thought to be primary contributors, including catecholamine surges and fluctuating hormone levels, among others. These changes can persist for 6 months post-partum. Women with PAMI due to SCAD tend to have left main or multivessel coronary involvement [8,12], and they often have associated reduced left ventricular function [17]. Of note, when SCAD is diagnosed, further imaging to evaluate for fibromuscular dysplasia or other arteriopathies should be pursued after delivery [19,20].

### 3.2. Coronary Embolism or Thrombosis

Pregnancy is an intrinsically hypercoagulable state. This can lead to the formation of spontaneous thrombi or emboli in the coronary arteries. Coronary thrombosis and coronary embolism cause an estimated 17–21% [5,8] and 3–4% [21] of ACS in pregnancy, respectively. In a study by Shibata et al., coronary emboli formation was most commonly associated with atrial fibrillation (73%), underlying cardiomyopathy (25%), and valvular disease (15%) [21]. Other predisposing factors include Kawasaki disease, autoimmune disorders, and systemic hypercoagulable states [13,14]. For women with both coronary thrombi and emboli, it is reasonable to consider anticoagulation and post-partum hypercoagulability workup if appropriate [5,12].

### 3.3. Coronary Vasospasm

Coronary vasospasm causes an estimated 2–5% of pregnancy-associated ACS [7,8]. It is hypothesized that some of the physiologic changes of pregnancy, including increased systemic renin and angiotensin levels, as well as endothelial dysfunction, may predispose to coronary vasospasm [22,23,24].

### 3.4. Normal Coronary Arteries

Approximately 11–29% of ACS in pregnancy is associated with normal coronary arteries [5,8,12].

### 3.5. Atherosclerotic Coronary Artery Disease

Approximately 27–43% of PAMI is caused by atherosclerotic CAD [5,8,12]. In one of the few prospective studies available, Baris et al. showed that women with underlying atherosclerotic disease during pregnancy are significantly older; have a higher BMI; and have a higher prevalence of traditional CAD risk factors, including smoking and hypertension [25]. As women enter pregnancy at older ages, the prevalence of atherosclerosis during pregnancy is expected to rise.

## 4. Risk Factors

There are numerous risk factors for ACS in pregnancy. One of the strongest risk factors is advanced maternal age (>30 years of age) [5,7,8]. Women with traditional atherosclerotic risk factors, including hypertension, hyperlipidemia, and type 2 diabetes, are also at higher risk of PAMI, but these risk factors are present in only a minority of women [5,6,8]. There are also sex-specific risk factors for PAMI, including preeclampsia [6] and gestational diabetes [12,16]. Black women have higher rates of PAMI than white women, as do women of lower socioeconomic status [5,6,7]. Though data are limited, Hispanic women appear to have lower rates of PAMI than Black women [6,26]. One analysis from a large national US-based database showed that women who developed PAMI were 10 times more likely to have risk factors than women who did not (66.1 per 100,000 cases compared with 5.2 per 100,000 cases without, respectively) [16]. Women with known risk factors for cardiovascular disease and prior pregnancy complications should consider pre-pregnancy counseling given their higher risk of ACS during pregnancy [14].

## 5. Evaluation and Management of ACS in Pregnancy and Post-Partum Period

### 5.1. Diagnosis

The diagnosis of ACS in pregnancy relies on the same principles as in the non-pregnant patient, namely, anginal symptoms, changes on the electrocardiogram (ECG), and elevated cardiac biomarkers. Women may present with typical or atypical anginal chest symptoms during pregnancy. PAMI tends to occur most often in the third trimester or early post-partum period, and NSTEMI is more common in pregnancy than STEMI [16].

Although there are subtle ECG changes during normal pregnancy, including left axis deviation, T-wave inversions, and Q waves, ST segment elevation is never normal. The same ECG criteria used to diagnose STEMI and NSTEMI in non-pregnant patients should also apply to pregnant patients. Furthermore, although large-scale studies examining cardiac biomarkers in pregnancy are lacking, elevated serum troponin levels during pregnancy suggest underlying myocardial ischemia and should be evaluated further [27,28].

### 5.2. Diagnostic Coronary Angiography

In patients with pregnancy-associated ACS, coronary angiography is the diagnostic and therapeutic gold standard. In patients who present with STEMI or hemodynamically unstable NSTEMI, coronary angiography should be offered regardless of pregnancy status [2,13,29,30].

In low-risk patients with NSTEMI who have no evidence of ongoing ischemia, LV systolic dysfunction, or hemodynamic instability, expert consensus recommends consideration of ischemia-guided medical management without invasive angiography [14,31]. For non-invasive testing, stress testing is not typically used during pregnancy, but coronary computed tomography angiography (coronary CTA) can be safely used if necessary [32]. If coronary CTA is pursued during pregnancy, fetal and maternal radiation protection is essential, and protocols can be used to reduce the radiation dose [32]. Iodinated contrast does cross the placenta, however [33]. Though data are limited, there are no available data to suggest that iodinated contrast is teratogenic, and the American College of Radiology does not recommend avoiding its use in the pregnant patient if it is necessary [34].

If invasive coronary angiography is pursued, appropriate maternal and fetal protection, including lead shielding and short fluoroscopy times, are essential. The fetal risk of radiation is inversely related to gestational age, with the highest risk of fetal injury prior to 20 weeks gestational age [2,29,35]. Fetal risk of radiation is also inversely related to radiation dose, with no evidence of fetal injury or loss when radiation exposure is <50 mGy [29,35]. Most diagnostic coronary angiograms and percutaneous coronary interventions occur with a radiation dose significantly below this level [14,29,30]. Of note, thrombolysis carries numerous risks during pregnancy, including maternal hemorrhage, fetal hemorrhage and death, and preterm birth, and should only be offered if coronary angiography is both indicated and unavailable [36,37,38]. The risk of significant maternal bleeding after thrombolysis is especially associated with delivery [38,39,40]. Despite the radiation risks of coronary angiography, there are clear benefits to proceeding with cardiac catheterization when appropriate, as women with MI who undergo coronary angiography have lower in-hospital mortality than women who do not pursue invasive therapy [16]. Before any invasive procedure or thrombolysis is performed in a pregnant woman, a multidisciplinary cardio-obstetrics team should be aware and available should there be maternal hemodynamic decompensation [36].

## 6. Management

### 6.1. Medical Management

The medical management of ACS during pregnancy is similar to that in the non-pregnant patient but may require slight modification. Full-dose aspirin (ASA 325 mg) can be used up to 32 weeks gestational age, and ASA 81 mg can be safely used throughout pregnancy [13,41]. Heparin does not cross the placenta and so is the preferred anticoagulant, though its use should be discontinued prior to delivery [41]. Nitrates can be used, but maternal hypotension should be avoided, especially due to the risk of placental hypoperfusion [8]. If dual antiplatelet therapy (DAPT) is required after PCI, clopidogrel is the preferred P2Y12 inhibitor [41]. The use of P2Y12 inhibitors must be discontinued 7 days prior to neuraxial anesthesia, but this is not the case for ASA. Heparin, in this context, cannot be used in lieu of DAPT. Beta blockers are safe during pregnancy, and metoprolol is preferred [41]. Angiotensin-converting enzyme inhibitors (ACEi) and angiotensin receptor blockers (ARB) should be avoided [41]. Statins are contraindicated [41].

### 6.2. Invasive Management

The decision to address obstructive or intervenable coronary disease in a pregnant patient should be guided by many factors. These include the mechanism of ACS, gestational age, maternal and fetal clinical status, coronary anatomy, and availability of mechanical circulatory support (MCS), among others.

For obstructive lesions causing STEMI that are amenable to percutaneous coronary intervention (PCI), proceeding with PCI is appropriate. The choice of whether to use bare metal stent (BMS) or drug eluting stent (DES) is an evolving space, especially in the context of minimizing the duration of DAPT. DAPT duration in pregnancy is a critical issue, given both the increased bleeding risk associated with DAPT and the importance of stopping P2Y12 inhibition prior to neuraxial anesthesia. As such, DAPT in the third trimester can be challenging, especially given that the safety profile of intravenous P2Y12 inhibitors such as cangrelor is unknown in pregnancy [13]. For these reasons, BMS were historically used in pregnancy [8,12], and available data support the safety of their use [8,12]. As the recommended DAPT duration in DES shortens, however, BMS is likely to fall increasingly out of favor. This is especially true given that DES have been shown to be used safely in pregnancy [8,42,43]; the safety and success profile of DES in the non-pregnant patient is favorable when compared to BMS [8,43]. Newer generation DES are also approved by the United States Food and Drug Administration (FDA) for DAPT duration as short as 28 days [44], which may be advantageous given the potential limitations of DAPT use at the time of peri-delivery anesthesia.

For STEMI without evidence of obstructive lesions, further management should be guided by the presumed underlying cause. In patients thought to have thromboembolism, aspiration thrombectomy, balloon angioplasty, and antithrombotic therapy may all be considered [45].

If SCAD is thought to be the etiology, a conservative approach with medical management is preferred, as PCI in SCAD is associated with high procedural complication rates and limited success and should be avoided unless necessary [8,19,46,47,48]. Regardless of pregnancy status, if a patient with presumed or confirmed SCAD presents with left main or multivessel coronary involvement, impaired systolic function, shock, or hemodynamic instability, emergent PCI or coronary artery bypass graft (CABG) may be required. Peripartum SCAD is independently associated with high-risk features and multivessel involvement [48,49], and further invasive evaluation may be required. Thrombolysis is not recommended in SCAD given the risk of dissection flap propagation and association with high fetal and maternal mortality [14,48].

Surgical revascularization with CABG can be considered in pregnancy for patients with ongoing ischemia who are unresponsive to medical interventions, are poor PCI candidates, and would otherwise meet the same indications for surgical revascularization as in the non-pregnant patient. Though data are limited, CABG during pregnancy is highly morbid, with maternal mortality of 1.7–3% [50] and fetal mortality up to 20% [50]. Some of this mortality is associated with cardiopulmonary bypass (CPB) alone, with maternal mortality occurring in 3–15% of cases and fetal mortality occurring in 16–33% [51]. Even in patients who survive, the risks of CPB during pregnancy are significant and include utero-placental hypoperfusion, premature delivery, fetal hypoxia, and neonatal respiratory distress syndrome, among others [52,53]. The best outcomes for surgical revascularization are when CABG is performed at 13–26 weeks gestation [14]. When gestational age is >28 weeks, emergent delivery prior to CABG should be considered [14]. If CABG is pursued during pregnancy, there are a number of specific considerations. If SCAD is the indication for CABG, native vessel healing may cause the grafts to become atretic or cause competitive flow [46]. Furthermore, though rare, if SCAD is associated with FMD, the left internal mamillary artery may be affected and become a sub-optimal graft choice.

### 6.3. Management of Post-MI Complications including Shock

Cardiogenic shock in the peripartum period necessitates early identification and optimal management in order to reduce maternal–fetal adverse outcomes. In the general population, ACS is complicated by cardiogenic shock 5–12% of the time [54], while in PAMI, the incidence of shock is higher. Cardiogenic shock in this population is likely more common for numerous reasons, one of which is the increased incidence of reduced LV systolic dysfunction. In one review of 150 cases of PAMI, more than half of women had new systolic dysfunction (LV ejection fraction (LVEF) < 40%), with 24% of women having LVEF ≤ 30%, and 9% having LVEF ≤ 20% [8]. In this population, 38% of patients developed cardiogenic shock [8]. Among all cases of cardiogenic shock in pregnancy, peripartum cardiomyopathy (PPCM) is the most common etiology, followed by acute MI, pulmonary embolism (PE), amniotic fluid embolism (AFE), and other pre-existing cardiac conditions [55,56,57]. In a large national inpatient sample study of over 53 million pregnancy-related hospital admissions, 2044 women developed cardiogenic shock, most of which occurred in the post-partum period (56.8%) [56]. Additionally, 56.3% of the cohort had PPCM-related cardiogenic shock, 13% had PAMI, 7% PE, and 4% AFE [56].

**Figure 1:**
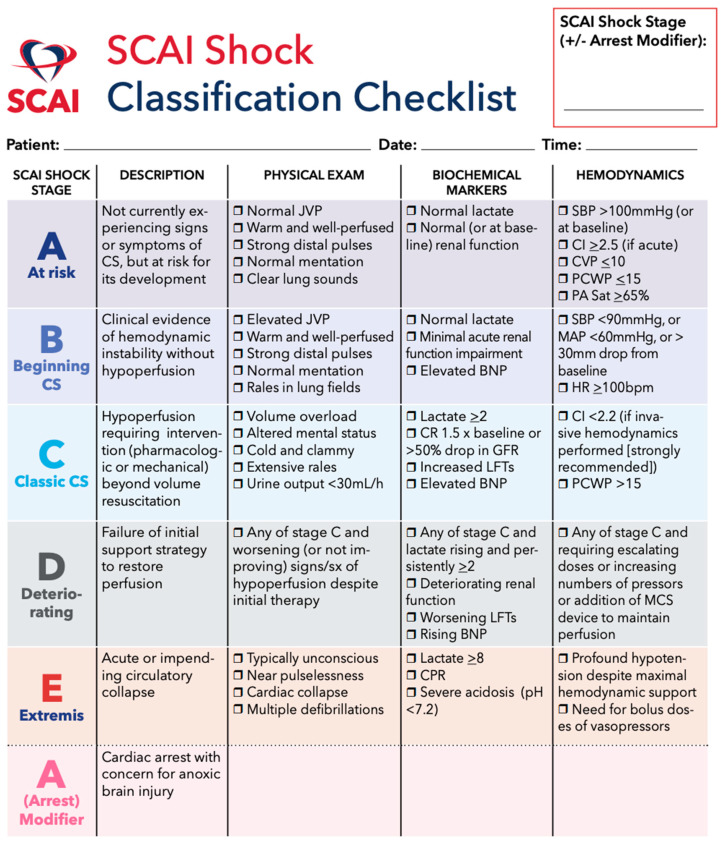
The Society for Cardiovascular Angiography & Interventions (SCAI) Shock Classification Checklist for the assessment of cardiogenic shock [59].

Optimal management of cardiogenic shock involves rapid diagnosis utilizing physical exam, biomarkers, echocardiography, and invasive hemodynamics (Figure 1). While most cases of PAMI-related cardiogenic shock are due to the sequelae of LV dysfunction, echocardiography is useful for the evaluation of mechanical complications, including papillary muscle rupture and ventricular septal defect (VSD). An early hemodynamic profile assessment may define the shock phenotype and guide management [58]. While initial medical therapy with inotropes and vasopressors are first line, consideration of mechanical circulatory support (MCS) is often necessary.

Mechanical circulatory support, including intra-aortic balloon pump, Impella, and veno-arterial extracorporeal membrane oxygenation (VA-ECMO), should be considered when failure of medical therapy occurs. Early deployment of MCS decreases mortality, particularly when initiated within 5 days of onset of shock [56]. However, there is a paucity of data to guide optimal device selection. Intra-aortic balloon pumps may be appropriate in PAMI; however, few data support their use [60]. In terms of Impella use, a case series of 15 peripartum women with PPCM who received Impella support had a survival rate of 86.7% [61].

VA-ECMO provides cardiopulmonary support and may be utilized in pregnancy for biventricular support. Unlike Impella, VA-ECMO may be initiated rapidly at the bedside and is the optimal MCS strategy during cardiac arrest. The largest systematic review to date of 358 peripartum ECMO runs demonstrated a 75.4% 30-day and 74.3% 1-year maternal survival [62]. Furthermore, among the patients receiving VA-ECMO support for PPCM, survival was 78.3%. Additionally, among the cohort, 35 deliveries occurred while on ECMO, of which 79.4% of women and 56.3% of fetuses survived [62]. While ECMO survival among pregnant women is robust, all MCS decision making is best made in conjunction with a cardio-obstetrics and shock team.

## 7. Delivery and the Post-Partum Period

When feasible, decisions around timing of delivery in patients who have had pregnancy-associated ACS should be guided by a multidisciplinary cardio-obstetrics team [13,14]. Unless there are clinical indications for urgent or emergent delivery, practice guidelines and expert consensus recommend delaying delivery until at least 2 weeks after PAMI to prevent maternal complications [12,14]. Though data are limited, expert consensus recommends proceeding with vaginal delivery unless there is maternal hemodynamic compromise or fetal indications [12,63]. Obstetrical considerations to optimize maternal cardiac output during delivery include minimizing Valsalva efforts [13,64] and the potential use of instrumental assistance [13,64]. For women whose hemodynamics may be especially tenuous at the time of labor and delivery, including women with LV systolic dysfunction, invasive hemodynamic monitoring (e.g., with Swan-Ganz catheter) may also be safely considered [13,65]. In general, however, expert consensus is that invasive hemodynamic monitoring with a Swan-Ganz catheter is rarely appropriate [14]. Appropriate anesthesia may also help to reduce hemodynamic fluctuations. Anesthetic choices at the time of delivery are dictated by numerous factors, including hemodynamic stability, patient preference, and obligate maternal medications including DAPT [13,64]. Data and guideline consensus are limited regarding optimal management after termination in women with PAMI, though expert opinion suggests treating the period after termination as the post-partum period and caring for women accordingly on a case-by-cases basis [13,36]. Most women who deliver after PAMI are monitored in an intensive care setting [13].

Women may also present with new ACS in the post-partum period. Any non-pregnant woman of childbearing age who presents with chest discomfort should be asked about recent pregnancy as the hypercoagulability of pregnancy persists for weeks after delivery [66]. MI-associated mortality appears to occur less in the post-partum period than during pregnancy [6,12], though the etiologies of MI during pregnancy and in the post-partum period are similar [8,12]. SCAD is the most common cause of ACS in the post-partum period [8]. The baseline electrocardiogram normalizes after delivery, and any ST segment change should prompt further workup for underlying ischemia. Biomarkers are frequently elevated after delivery in pregnancies complicated by preeclampsia and gestational hypertension [67], though they may also be elevated in asymptomatic women in the post-partum setting [68]. Regardless, troponin elevation in the post-partum setting should always prompt further investigation. Because fetal safety is no longer a necessary consideration, decisions regarding diagnostic testing to further evaluate underlying ischemia should be similar to those in the non-pregnant patient [13,69].

Whether women with a history of PAMI may pursue future pregnancies is best determined through a shared decision-making, individualized approach prior to conception [13,14,36]. In general, LV function and etiology of PAMI are primary determinants of safety in future pregnancy. Women with severe LV dysfunction (LVEF < 30%) are generally not advised to pursue future pregnancy [14]. In women with PAMI due to SCAD, future pregnancy is generally contraindicated [19]. Though data are limited, available data suggest that recurrent SCAD affects 13–18% of women [70,71,72] with emerging evidence that subsequent pregnancy may not necessarily increase the risk for SCAD recurrence [70]. Many women with SCAD, however, do not become pregnant again [70,73]. For women without pregnancy contraindications and who wish to pursue subsequent pregnancy, expert consensus recommends waiting at least 12 months after MI and extensive preconception counseling [14]. An individualized, multidisciplinary approach to offering recommendations for future pregnancy is advised.

## 8. Conclusions

Though chest pain during pregnancy and in the post-partum period has a broad differential, ACS should always be considered. This is especially true given that ACS is more common in women during pregnancy than in similarly aged non-pregnant women. The principles of diagnosis and treatment of ACS during pregnancy and the post-partum period are similar to those in the non-pregnant patient, though numerous special considerations must be taken in order to optimally protect both the mother and her fetus. Complications of ACS during pregnancy, including cardiogenic shock, must be identified early and promptly managed in order to reduce maternal and fetal adverse outcomes. Importantly, considerations regarding timing and method of delivery must be guided by both maternal and fetal stability. The early, and close, engagement of a multidisciplinary cardio-obstetrics team is essential.

## Data Availability

Not applicable.
